# Is Persistent Post-COVID Headache Associated With Protein-Protein Interactions Between Antibodies Against Viral Spike Protein and CGRP Receptor?: A Case Report

**DOI:** 10.3389/fpain.2022.858709

**Published:** 2022-04-01

**Authors:** Esra Özkan, Özlem Celebi, Özlem Keskin, Attila Gursoy, Yasemin Gürsoy-Özdemir

**Affiliations:** ^1^Koç University Research Center for Translational Medicine, Istanbul, Turkey; ^2^Department of Neurology, School of Medicine, Koç University, Istanbul, Turkey; ^3^College of Engineering, Chemical and Biological Engineering, Koç University, Istanbul, Turkey; ^4^Department of Computer Science and Engineering, College of Engineering, Koç University, Istanbul, Turkey

**Keywords:** SARS-CoV-2, migraine, calcitonin gene-related peptide (CGRP), antagonist, post-COVID, spike protein, monoclonal antibody

## Abstract

**Background:**

After the acute pandemic of coronavirus disease 2019 (COVID-19), a wide variety of symptoms are identified under the term post-COVID syndrome, such as persistent headache. Post-COVID headache can be presented in a broad spectrum like headache attributed to systemic infection, chronification of already existing primary headache, or long-lasting, and also late-onset new daily persistent headache. Still, little is known about the pathophysiology of post-COVID headache, but activation of the trigeminovascular system may be one of the players.

**Case Report:**

Here, we present a case with a severe, long-lasting post-COVID headache and its sudden cessation with calcitonin gene-related peptide (CGRP) monoclonal antibody treatment.

**Conclusion:**

In our previous protein mimicry study, we have pointed at mimicry of virus spike protein and CGRP receptors. This mechanism may enlighten the current, common, and yet unsolved post-COVID headache cases.

## Highlights

- CGRP mAbs must be considered as a treatment option in drug-resistant post-COVID headache cases.
- The activation of the trigeminovascular system due to protein mimicry between CGRP receptors and antibodies against SARS-CoV-2 spike protein can enlighten the unsolved mechanisms of post-COVID cases.

## Introduction

Coronavirus disease 2019 (COVID-19) first emerged in China in December 2019 and was accepted as a global pandemic on 26 August 2020. A novel coronavirus, particularly the severe acute respiratory syndrome coronavirus 2 (SARS-CoV-2), is reported to be responsible for COVID-19 (1).

Similar to the course of other systemic viral infections, a mild headache can be categorized as “headache attributed to systemic infection” according to guidelines (2) is observed with COVID-19 (3). Again in acute phase of COVID-19, headache phenotyes resembling migraine and tension-type headache were documented (3). After the acute phase of COVID-19, some patients exhibited persistent headaches, could not fully recover, or reported a delayed onset headache (4), referred to as a post-COVID headache.

A post-COVID headache includes a complex spectrum of presentations, including chronification of an already existing migraine, late-onset new persistent headache, or migraine-like headache without a prior history. Furthermore, some patients exhibit additional symptoms like fatigue, insomnia, memory impairment, dizziness, etc. (4, 5). Thus, these different clinical presentations probably stem from distinct pathophysiological mechanisms and must be defined and treated accordingly.

Here, we present a patient with a severe, long-lasting post-COVID headache. Furthermore, we discuss alternative treatment options in post-COVID headache that is resistant to routine treatments and provide insights into possible mechanisms behind the cases resembling our case.

## Case Report

A patient who is a 44-year-old woman with no other comorbidities or drug use except a history of low-frequency episodic migraine (< 1 day/month) had a mild COVID-19 infection at the beginning of August 2021. The patient's symptoms related to COVID-19 were headache, fatigue, cough, and shortness of breath at the time of her visit to the outpatient clinic of another hospital. The COVID-19 diagnosis was confirmed with a positive RT-PCR assay test. A computerized lung tomography was taken to rule out pneumonia. The patient recovered from COVID-19 at home without a need for hospitalization. The patient's headache was characterized as a frontally located pressure-like feeling with mild intensity. But after 15 days, the patient's pain intensity due to headache increased and was associated with nausea but without vomiting. The pain was pulsatile, bilateral, and severe photophobia; eventually, phonophobia was added. The patient reported that this headache was different from her previous migraine attacks because its intensity was higher, and after 25 days, she was unable to carry out routine daily activities due to this headache. She started taking 2,000 mg of paracetamol and 150 mg of diclofenac sodium without any pain relief daily. The patient's pain was present as soon as she woke up, and she reported it got worse when lying. She took sick leave from work and was diagnosed at another hospital. Brain MRI and fundus examination were done to rule out secondary causes of headache and came out with normal results. The neurologist prescribed 32 mg of methylprednisolone and 500 mg of acetazolamide to be taken daily in the outpatient clinic.

The patient first visited our headache outpatient clinic at the end of September 2021 to discuss persistent headaches. Her severe daily headache persisted, although she was taking several medications as well as methylprednisolone. At the time of evaluation, she used 600 mg of paracetamol, 150 mg of diclofenac sodium, and 500 mg of chlorzoxazone daily. At that time, the methylprednisolone dose gradually decreased up to 8 mg because of non-responsiveness. She also exhibited fatigue, insomnia, and loss of memory. Her neurological examination was normal, and repeated fundus examination did not detect papilledema. Her previous MRI was checked, and secondary causes of headaches were ruled out. According to the visual analog scale, she reported that her headache was 10 out of 10 and needed immediate pain relief. Loading dose (240 mg) of subcutaneous galcanezumab, calcitonin gene-related peptide receptor (CGRP) monoclonal antibody (mAb), was prescribed. During follow-up, her headaches abruptly decreased in both intensity and frequency within 2 days with the CGRP mAb treatment. She reported that her headache decreased to a scale of 2-3 out of 10. During the follow-up, the intensity of her headaches increased gradually to 5 out of 10 in 3 weeks. As a result, the second dose (120 mg) was prescribed, and the intensity of headaches decreased to 1–2 out of 10 within 2 days again. Monthly, 120 mg of CGRP mAb treatment was planned during follow-up. She was headache-free when last seen after the fourth month's dose of galcanezumab.

## Discussion

This case report presented a successful and immediate response of severe post-COVID headache to CGRP mAb treatment. This case is noteworthy in a couple of ways. First, the safety and efficacy of CGRP mAbs for post-COVID headache have been discussed before (6), but to our knowledge, this is the first case documented. From treatment to understanding, we also had an opportunity to discuss using this case a new route for activating the trigeminovascular system in infectious diseases, which is not rare in medicine.

The diagnosis of our patient's headache was considered as headache attributed to systemic viral infection at the first 2 weeks of COVID-19 diagnosis (2). But later on, the headache fulfilled the criteria of migraine without aura which did not end in 72 h. Although the overuse of abortive medications helped in resolving the headache at the end of 1 month, the best fit diagnosis was status migrainosus associated with fatigue, insomnia, and memory impairments.

Headache during the acute phase of COVID-19 is shared and reported between 34 and 75% of patients (7, 8). However, nearly one-third of patients experienced long-lasting symptoms (4, 8). Headache attributed to systemic infection diagnosis could not reveal the broad spectrum of post-COVID headache cases (2). Clinically, complicated presentation as migraine-like features, long duration, late-onset, and low responsiveness to medication suggests the role of different pathophysiological mechanisms. Although patients with a history of primary headaches are more likely to have a complicated presentation in the acute phase and longer duration, half of the patients with this phenotype have no history of primary headaches (8).

Fever and inflammatory cytokines are proposed as responsible factors for headaches attributed to systemic infection (9). Interestingly, studies showed a dissociation between fever or higher cytokine levels with post-COVID headache cases (7, 8). On the other hand, migraine-like features, including photophobia/phonophobia and responsiveness to triptans in patients without migraine history, could be explained with activation of trigeminovascular system (5). Most probably, this activation was triggered by direct invasion of the virus rather than systemic effects. Supporting this hypothesis, the proteins of SARS-CoV-2 are shown at the branches of the trigeminal nerve and trigeminal ganglion (10). This finding can explain the acute phase of headache, but there is no explanation other than systemic effects for delayed, post-infection headaches. In COVID-19, it is very well known that immunological reaction to the virus causes long-lasting problems rather than direct effects of the virus.

The role of CGRP is well-known in migraine pathogenesis. Moreover, interleukin (IL)-6 and tumor necrosis factor-alpha (TNF-a) are known to increase CGRP levels (11). CGRP receptors are also found with the angiotensin-converting enzyme and two receptors that SARS-CoV-2 binds for entering cells in the neurons of trigeminal ganglion (12). However, the lack of evidence about the positive correlation between post-COVID headache and inflammatory parameters raises the need to clarify the alternative mechanisms rather than the elevation of CGRP levels, such circumstance is what was explained above and one study finding reported lower CGRP levels in COVID-19 patients (13).

**Figure 1:**
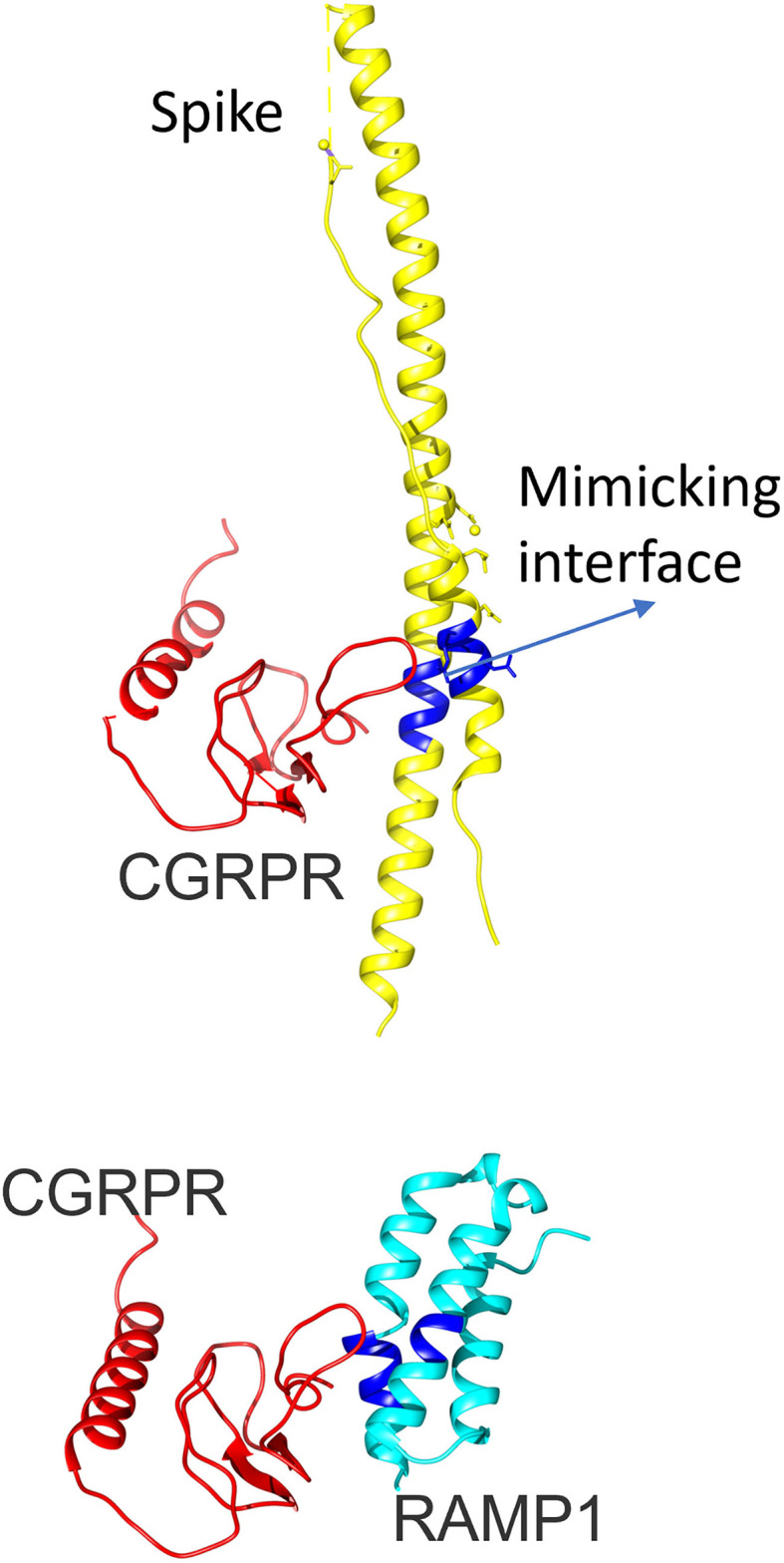
The 3D configuration of calcitonin gene-related peptide-receptor activity modifying protein 1 (CGRP-RAMP1) heterodimer and severe acute respiratory syndrome coronavirus 2 (SARS-CoV-2) spike protein. The upper panel shows the 3D configuration of the CGRP receptor (red) and SARS-CoV-2 spike protein (yellow). RAMP1 is added in the bottom figure in turquoise (CGRP-RAMP1 heterodimer, PDB ID: 3n7p, chains B and F). The mimicking interface with spike protein is highlighted with blue color. CGRP, Calcitonin gene-related peptide; RAMP1, Receptor activity modifying protein 1; SARS-CoV-2, severe acute respiratory syndrome coronavirus 2.

We wanted to discuss another possible mechanism for trigeminovascular activation as direct interaction of CGRP receptor with SARS-CoV-2 or antibodies against SARS-CoV-2. Our group reported in an interface mimicry study that the CGRP receptor has structural mimicry to spike protein of SARS-CoV-2 (14) (Figure 1). Furthermore, another group also demonstrated possible protein mimicry of spike protein with CGRP receptor and also receptor activity modifying protein 1 (RAMP1) (15). This mimicry may lead to a long-lasting reaction of antibodies in the body because of their resemblance with CGRP and its receptors, hence the post-COVID headaches. Furthermore, this may explain the higher frequency of headaches and why a persistent headache occurs in COVID-19 specifically rather than other viral systemic infections. Sudden cessation of the persistent severe headache of the patient treated with CGRP mAb may suggest activation of this pathway to provide indirect evidence for this pathophysiological mechanism, at least in some post-COVID persistent headache syndromes. Detailed molecular studies are needed to prove this possibility.

Our patient experienced a worsening after an excellent initial response to the treatment. The reason may be that the circulating antibodies against spike protein continue to interact with the CGRP receptor. Hence, it should be addressed if CGRP receptor antagonists like gepants and eranumab have better performances in treatment (16). Furthermore, since the RAMP1 domain is also shared in the Amylin subtype 1 receptor (AMY1) receptor complex (16), the possible clinical outcomes of AMY1 receptor activation due to spike protein mimicry should be considered in future studies. Finally, large-scale randomized controlled trials are required to study the efficacy of CGRP antagonist or therapy with a CGRP mAb in treating post-COVID headaches.

## Conclusion

Extensive data based on clinical observations are accumulating about the broad spectrum of post-COVID headaches. We present a severe persistent, long-lasting post-COVID headache, which is successfully treated with CGRP mAb. We also suggest a new possible route for activation of the trigeminovascular system *via* direct interaction of spike protein of SARS-CoV-2 or antibody against it with CGRP receptor.

## Data Availability Statement

The raw data supporting the conclusions of this article will be made available by the authors, without undue reservation.

## Ethics Statement

Ethical review and approval was not required for the study on human participants in accordance with the local legislation and institutional requirements. The patients/participants provided their written informed consent to participate in this study. Written informed consent was obtained from the individual(s) for the publication of any potentially identifiable images or data included in this article.

## Author Contributions

YG-Ö: study concept and design and revising it for intellectual content. ÖC and YG-Ö: acquisition of data. EÖ, ÖC, and YG-Ö: analysis and interpretation of data and drafting of the manuscript. ÖK and AG: establishment of protein mimicry of CGRP and spike protein. All authors final approval of the completed manuscript.

## Conflict of Interest

The authors declare that the research was conducted in the absence of any commercial or financial relationships that could be construed as a potential conflict of interest.

## Publisher's Note

All claims expressed in this article are solely those of the authors and do not necessarily represent those of their affiliated organizations, or those of the publisher, the editors and the reviewers. Any product that may be evaluated in this article, or claim that may be made by its manufacturer, is not guaranteed or endorsed by the publisher.

## Abbreviations

CGRP: Calcitonin gene-related peptide
COVID: Coronavirus disease
MRI: Magnetic resonance imaging
mAb: Monoclonal antibody
RAMP1: Receptor activity modifying protein 1
SARS-CoV-2: Severe acute respiratory syndrome coronavirus 2.
