# Supplementary material for: Is Persistent Post-COVID Headache Associated With Protein-Protein Interactions Between Antibodies Against Viral Spike Protein and CGRP Receptor?: A Case Report
Source: Front Pain Res (Lausanne). 2022 Apr 1;3:858709. doi: 10.3389/fpain.2022.858709 (PMC9011137; doi:10.3389/fpain.2022.858709)
Supplement: Supplementary file 1 [file Table_1.DOCX]

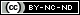
** CARE Checklist (2013) of information to include when writing a case report**

| **Topic** | **Item** | **Checklist item description** | **Reported on Page** |
| --- | --- | --- | --- |
| **Title** | **1** | The words “case report” should be in the title along with what is of greatest interest in this case . . . . . . . . . . . . . . . . . . . . . . | **1** |
| **Key Words** | **2** | The key elements of this case in 2 to 5 key words . . . . . . . . . . . . . . . . . . . . . . . . . . . . . . . . . . . . . . . . . . . . . . . . . . . . . . . | **2** |
| **Abstract** | **3a** | Introduction—What is unique about this case? What does it add to the medical literature? . . . . . . . . . . . . . . . . . . . . | **3** |
|  | **3b** | The main symptoms of the patient and the important clinical findings . . . . . . . . . . . . . . . . . . . . . . . . . . . . . . . . . . . . . . | **3-5** |
|  | **3c** | The main diagnoses, therapeutics interventions, and outcomes . . . . . . . . . . . . . . . . . . . . . . . . . . . . . . . . . . . . . . . . . . . | **4-5** |
|  | **3d** | Conclusion—What are the main “take-away” lessons from this case? . . . . . . . . . . . . . . . . . . . . . . . . . . . . . . . . . . . . . | **7** |
| **Introduction** | **4** | Brief background summary of this case referencing the relevant medical literature . . . . . . . . . . . . . . . . . . . | **3** |
| **Patient Information** | **5a** | Demographic information (such as age, gender, ethnicity, occupation) . . . . . . . . . . . . . . . . . . . . . . . . . . . . . . | **3-4** |
|  | **5b** | Main symptoms of the patient (his or her chief complaints) . . . . . . . . . . . . . . . . . . . . . . . . . . . . . . . . . . . . . . . . . | **3-4** |
|  | **5c** | Medical, family, and psychosocial history including co-morbidities, and relevant genetic information . . | **3-4** |
|  | **5d** | Relevant past interventions and their outcomes . . . . . . . . . . . . . . . . . . . . . . . . . . . . . . . . . . . . . . . . . . . . . . . . . . . | **3-4** |
| **Clinical Findings** | **6** | Describe the relevant physical examination (PE) findings. . . . . . . . . . . . . . . . . . . . . . . . . . . . . . . . . . . . . . . . . . . | **4-5** |
| **Timeline** | **7** | Depict important milestones related to your diagnoses and interventions (table or figure) . . . . . . . . . . . . | **-** |
| **Diagnostic Assessment** | **8a** | Diagnostic methods (such as PE, laboratory testing, imaging, questionnaires). . . . . . . . . . . . . . . . . . . . . . . | **4-5** |
|  | **8b** | Diagnostic challenges (such as financial, language, or cultural) . . . . . . . . . . . . . . . . . . . . . . . . . . . . . . . . . . . . . | **4-5** |
|  | **8c** | Diagnostic reasoning including other diagnoses considered . . . . . . . . . . . . . . . . . . . . . . . . . . . . . . . . . . . . . . . . | **4-5** |
|  | **8d** | Prognostic characteristics (such as staging in oncology) where applicable . . . . . . . . . . . . . . . . . . . . . . . . . . | **-** |
| **Therapeutic Intervention** | **9a** | Types of intervention (such as pharmacologic, surgical, preventive, self-care) . . . . . . . . . . . . . . . . . . . . . . . . . . . . . . . | **4-5** |
|  | **9b** | Administration of intervention (such as dosage, strength, duration) . . . . . . . . . . . . . . . . . . . . . . . . . . . . . . . . . . . . . . . . | **4-5** |
|  | **9c** | Changes in intervention (with rationale) . . . . . . . . . . . . . . . . . . . . . . . . . . . . . . . . . . . . . . . . . . . . . . . . . . . . . . . . . . . . . . . | **4-5** |
| **Follow-up and**  **Outcomes** | **10a** | Clinician- and patient-assessed outcomes . . . . . . . . . . . . . . . . . . . . . . . . . . . . . . . . . . . . . . . . . . . . . . . . . . . . . . . . . . . . . | **4-5** |
|  | **10b** | Important follow-up test results . . . . . . . . . . . . . . . . . . . . . . . . . . . . . . . . . . . . . . . . . . . . . . . . . . . . . . . . . . . . . . . . . . . . . . | **5** |
|  | **10c** | Intervention adherence and tolerability (How was this assessed?) . . . . . . . . . . . . . . . . . . . . . . . . . . . . . . . . . . . . . . . . . | **-** |
|  | **10d** | Adverse and unanticipated events . . . . . . . . . . . . . . . . . . . . . . . . . . . . . . . . . . . . . . . . . . . . . . . . . . . . . . . . . . . . . . . . . . . | **-** |
| **Discussion** | **11a** | Discussion of the strengths and limitations in the management of this case . . . . . . . . . . . . . . . . . . . . . . . . . . . . . . . . . | **5** |
|  | **11b** | Discussion of the relevant medical literature. . . . . . . . . . . . . . . . . . . . . . . . . . . . . . . . . . . . . . . . . . . . . . . . . . . . . . . . . | **5-6** |
|  | **11c** | The rationale for conclusions (including assessment of possible causes) . . . . . . . . . . . . . . . . . . . . . . . . . . . . . . . . . . . | **5-7** |
|  | **11d** | The main “take-away” lessons of this case report . . . . . . . . . . . . . . . . . . . . . . . . . . .. . . . . . . . . . . . . . . . . . . . . . . . . . | **7** |
| **Patient Perspective** | **12** | Did the patient share his or her perspective or experience? (Include whenever possible) . . . . . . . . . . . . . . . . . . . . . . | **-** |
| **Informed Consent** | **13** | Did the patient give informed consent? Please provide if requested . . . . . . . . . . . . . . . . . . . . . . . . . . . . . . . . . . . . . . | **Yes**  **No** |
